# A Letter from Paris

**Published:** 1875-05-01

**Authors:** C. C. Matteson

**Affiliations:** Paris


### A LETTER FROM PARIS.

EDITORS MEDICAL EXAMINER: Since my last letter the number of Parisian medical journals, already great, has been increased by the appearance of the *Annales des Maladies de l' Orielle et du Larynx*. This journal is to appear every two months, and is published by MM. Ladreit de Lacharriere, physician in chief at the National Institution for the Deaf and Dumb, Isambert, physician to the hospital Lariboisiere, and Krishaber, free professor of laryngeal pathology. It will be unique in its field of investigation in France, and the editors state in their opening article that their intention is to unite in the same publication the diseases that Otoscopy, Laryngoscopy, and Rhinoscopy permit to study, inasmuch as these diseases are united by continuity of tissue. The first number contains, among other articles, an essay on the classification of the diseases of the larynx and pharynx, by Dr. Isambert; an account of the disease of Meniere and vertigo in diseases of the ear, by Dr. Ladreit de Lacharriere, and an article on rhinoscopy by Dr. Krishaber. Altogether, the journal starts under very favorable auspices and will be welcomed by the profession at large, though more particularly by the specialists.

Dr. Vibert (of Puy) has commenced a series of articles in the *Journal de Therapeutique*, on hypodermic injections of morphia, that promises to be long continued, as during a dozen years, from his own account, his therapeutics seem to have been somewhat largely confined to the use of a hypodermic syringe with a solution of morphia. From the child of seven years to the patriarch of seventy, this mode of treatment was followed, and though space forbids his mentioning many of his cases, we find recorded a great variety of the ills that man is subject to, from erysipelas to aortic insufficiency, from metrorrhagia to asthma, all yielding under the benign influence of opium given in this manner. Yet amid all these records, that but repeat experience almost classic, we find one fact that promises to be of service in measuring the effect of morphia upon the system. This is found in his first article in the *Journal* for February 25th. From his long experience Dr. Vibert has found that unless the pupil is immobile, unchanging with the presence of a light, the patient is not fully under the influence of the drug. From the lessening mobility of the pupil the effects of the morphia can be studied with certainty, and from this action the writer states that the pupil has become for him "a veritable manometer of the action of the morphia," which indicates just when to stop the exhibition of the medicament. To illustrate, he gave a woman 8 milligrammes of morphia in order to quiet her during a violent attack of hepatic colic. Half an hour afterward the patient, who had not vomited up to that time, commenced to vomit violently, and had no diminution of her sufferings. The question was whether the susceptibility of the patient to morphia had caused this, or whether it was due to the hepatic colic. Finding the pupils still dilated, he gave another injection of 12 milligrammes. Twenty minutes afterward the pupil was two-thirds contracted, and remained so in the dark, but would still contract under the influence of a light. There was some relief, but it was only relative. Finally, the pupil still preserving about one-third of its contractility, half an hour after the second injection he gave another centigramme, which caused a permanent closure, nearly complete, of the pupil, and at the same time enough relief to permit the patient to sleep. "The modifications of the pupil had thus furnished me with precise indications for the progressive dose of the medicine, and by that had permitted me to determine it with an exactitude nearly mathematical."

It seems that the profession in France is to lend its voice against the Morse system of telegraphing, if we can judge from a report made by M. Onimus before the Societe de Biologie on March 20th. He had had occasion to observe in telegraph operators phenomena analogous to those found in Scrivener's Palsy. In one case the person had been employed as a telegraph operator for nineteen years, and ten years ago had commenced to have difficulty in manipulating his instrument. At first he found that the letters *s*, represented by three dots, *i*, represented by two dots, and *u*, represented by two dots and a dash, were no longer neatly made. At the same time he felt a sort of cramp in the hand while tracing these letters. The letter *d*, made by a dash followed by two dots, he could trace more easily than the letter *u*, which is represented by the reverse—two dots followed by a dash. The man accustomed himself to sending his dispatches by using his thumb alone. For two years he had no difficulty, but at the end of that time his thumb was affected in turn. For two months he was able to continue his work with the index and middle fingers. Finally he could only use his fist to operate with, and, the cramps returning even when he performed his work in this way, he was forced to stop. In addition to the cramps there was a trembling of the hand and forearm when attempting to operate. Frequently he was troubled with sleeplessness and occasional signs of cerebral excitement.

Inasmuch as these accidents only arise from the use of the telegraphic signs of Morse, M. Onimus concludes that it may be necessary to abandon that system.

The indefatigable Dr. Bouchut gave a public demonstration of his cerebroscopic facts on March 16th. The little amphitheatre of the Hopital des Enfants being properly darkened for the occasion, for an hour and a half he illustrated, by means of a reflecting lamp, the appearance of the retina and blood-vessels of the eye as seen in different diseases of the brain and spinal cord. Many of the lesions had been verified by post-mortem examination, and the majority of his illustrations were taken from cases that he had had in his service since 1862, when he commenced his investigations on this subject.

The conclusions that he has arrived at as to the actual value of this means of diagnosis are published in the *Gazette des Hopitaux*, under the title of "Cerebroscopic Review for 1874." After giving in detail the history of observations made upon more than thirty cases, he concludes that the utility of the ophthalmoscope for medical diagnosis is demonstrated as follows:

When there is hyperæmia or hyperæmic swelling of the optic nerve, we can diagnose the mechanical or inflammatory hyperæmia of the brain found in meningitis, cerebral hæmorrhage, effusions in the brain, and sometimes the diagnosis can be made of spinal diseases, ataxic or other.

When he finds œdema of the papilla added to the hyperæmia, Dr. Bouchut diagnoses an œdema of the meninges or an embarrassment of the cerebral circulation that may be determined by meningitis, by certain cerebral tumors, by cerebral hæmorrhage, etc.

When there is an anæmia of the nerve in the retina and in the choroid (nervo-retinienne et choroïdienne), we can diagnose the cerebral hæmorrhage of softening, and if the anæmia is complete, it is a certain sign of death. The arrest of the cerebral and cardiac circulation is shown by the emptyness of the vessels of the eye, and by the bloodless network of the choroid.

When there is an inflammation of the retina and the nerve (nervo-retinite optique), with a fatty exudation, we can diagnose a chronic meningo-encephalitis, an encephalitis that depends on cerebral tumors and the change in brain substance that accompanies these tumors.

When there are varicose veins and thrombi in the retina, there are thrombi in the meninges or in the sinuses.

Aneurisms seen in the arteries of the retina show that there are miliary aneurisms in the brain.

When there is simply an hæmorrhage in the retina, we can assert that there exists a compression of the brain from hæmorrhagic or other effusion, but if this hæmorrhage is accompanied by a fatty degeneration of the retina, it is because there is cerebral steatosis, which is the case in chronic albuminuria, leucocythemia, and diabetes mellitus.

When there is atrophy of the optic nerve, we can diagnose the presence of tumors of the brain and of cerebral or spinal sclerosis.

Finally, tuberculous granulations are never seen in the choroid without the existence of similar changes in the lungs and other organs. In regard to the white spots that he names tuberculous granulations, it has been advanced that they are not really that in structure. Referring to this point, he argues that be their structure what it may, their presence demonstrates clinically that which he states.

An incident has occurred that does not reflect very favorably upon the Parisian priesthood. The *Journal Official* had commenced to publish a series of articles by Dr. Bouchut, in which hypnotism was discussed. While mentioning the effect of long-continued fixation of the sight upon an object near at hand, the Doctor showed that many of the remarkable experiences of olden times could be thus explained. The wondrous exhibitions that were sometimes given of fowls thrown into a state of insensibility from being forced to gaze at a chalk-mark, could easily be accounted for from fatigue of the nerves, and in the same manner other manifestations of this nature could be brought from the domain of the supernatural to that of natural phenomena. These practical views brought upon the editor of the *Journal* a host of opposition from the clergy, who denounced the publication of such heretic views. Not being able to stand against the pressure brought to bear upon him, the editor referred the matter to Dr. Bouchut. The latter finally published a letter in the *Journal*, stating that though he intended no disrespect to the traditions of the church, inasmuch as the articles seemed to create a difficulty he would discontinue them in the *Journal Official*, mentioning, however, that they would be published elsewhere; that is, in the medical journals.

Last year Pajot, professor of obstetrics and diseases of women and children, did not give his regular course of lectures, his place being supplied by a professor agrege. However, his retirement from the lecture-room being universally regretted, in response to a petition signed by all the Internes in the hospitals, and other students to the number of several hundreds, he has again commenced a course of lectures in the summer session of the school, which began on the 15th of March. The enthusiasm with which he was greeted was something immense. The grand amphitheatre of the school was crowded to suffocation. Probably there were a thousand persons inside that could hear the lecture or at least see the lecturer, while the passage ways were crowded with those that could neither see nor hear, but who testified to their feelings by following up the applause from within by hearty, if erratic, demonstrations.

The attendance does not seem to diminish with succeeding lectures, and it was only the third day that I was able to get a seat where I could hear the lecturer. The professor demonstrated the diameters and axis of the pelvis, a subject not intrinsically funny, and yet, with his curious manners and unexpected illustrations, he kept his large audience amused and attentive, not permitting the interest to flag during the hour.

Dr. Pajot is pre-eminently a teacher, and has devoted the greater part of his life to teaching rather than to the real practical life of the profession.

In fact, it is narrated of him that when a lady would ask him to attend her during an approaching confinement, he would invariably reply, "With the greatest pleasure, madame, if I do not happen to be lecturing at that time!" It is not difficult to believe that under such circumstances his private practice remained very limited.

The question of the use of chloroform in natural labor has been very hardly contested in France, and, in consequence of a paper published last year by Dr. Campbell upon the benefit of the use of chloroform under these circumstances, as illustrated by a large number of cases in his own practice, Prof. Pajot has written an article which is very severe in its opposition to such a course. It first appeared in the *Annales de Gynecologie* for January, and has since been reprinted in pamphlet form. The writer examines the question under three propositions: How do they give chloroform in natural labor? When do they give it? What are the results that they obtain?

The first question he discusses by quoting passages from the work of Dr. Campbell, to show that either they do not give enough of the chloroform to produce an anæsthesia, or that, producing the full effect, they do it at the risk of the life of the patient, as the accoucheur cannot watch the face of the mother and the descent of the child at the same time. In the latter case he accuses the supporters of this procedure of negligence. In the former case, he says they "are not serious." "It is homœopathic."

In regard to the second question, of when they give the chloroform, he states that it is only at the third period of expulsion, that being very painful, the *true* anæsthesia can be given with benefit, and then they are deprived of the possibility of directing the efforts of the mother. The advocates of this procedure do not give chloroform during the last hours of dilatation, the part of labor worst borne, but when, outside of the dangers, the indication for true anæsthesia is found.

The third question concerning the results of the anæsthesia of the advocates of chloroform in natural labor gives Professor Pajot an opportunity to show his talent for sarcasm that he does not fail to improve. He attacks them upon the ground that the pretended semi-anæsthesia is only a delusion. The indications that give warning of the approach of a period of insensibility are mentioned in this way by the advocates of this procedure: "There is a roaring in the ears that exists in nearly all the cases, and which, when it exists, appears to us to indicate that they have reached a state of semi-insensibility." Here is an opportunity that the Professor seizes with avidity, and he writes of it: "So here is the indication, precise, scientific! They ask the woman, 'Have you a noise in the ears?' 'No, sir.' 'Then you are not semi-insensible.' 'And now?' 'Yes, sir.' 'Good, you are now semi-insensible.' This recalls involuntarily the famous dialogue: 'Are you deaf and dumb?' 'Yes, sir.' 'Very good.'"

The statement that no accidents have yet resulted from the use of chloroform in this semi-anæsthesia he says should occasion the same amount of astonishment as when we fail to see fatal accidents from the use of bread pills. He contends that this means is good as a temporizer, but has no other usefulness. To illustrate this point, he narrates an experience of his own in temporizing, that I trust I shall be pardoned for translating, as it gives a good idea of the style of Prof. Pajot:

"I delivered some fifteen years ago a primipara, granddaughter of a dowager of seventy-five years. This aged dame, person of grand airs, as noble as dry, and as dry as impertinent—rare circumstance, for old families are generally of great politeness to us poor wretches, uncouth but useful — this respectable personage assisted at the labor. The expulsion advanced but slowly, the vagina being narrow and rigid, the perineum and the vulva resisting. The good but interrupted contractions discouraged the young woman. 'My dear,' said the dowager to me (it was the first time she had ever seen me), 'one of my friends has a medal that is good for delivering all women. Shall I go for it? 'Why not, madame,' said I, with deference, only too glad for this unexpected aid. The carriage departed, and half an hour afterward, when they announced the return of the noble grandmother, I gave the patient twenty-five centigrammes of ergot prepared in advance. The medal was placed on the breast of the girl, and twenty minutes afterward the labor was ended. During an hour the dowager deafened us concerning the suddenness of this miracle, and, head under the knife, she would have proclaimed the virtues of this talisman. It has served many times since; perhaps not always with such wonderful success."

Prof. Pajot concludes his article with several propositions on the use of chloroform in natural labor, as he judges it:

*True anæsthesia* applied to natural labor is a serious and scientific proceeding that can be discussed. Its dangers and disadvantages appear to us greater than its advantages.

The pretended *semi-anæsthesia* is as useless as inoffensive. It has nothing scientific or serious about it. It can be placed alongside those other dilatory means that act on the imagination of the woman, and that gain time, when in natural labor there is need of nothing else. They can make women think they would have suffered more had it not been for the semi-anæsthesia. Chloroform will be for the woman in child-bed like Providence, that it is always necessary to thank when one breaks one leg—both might have been broken.

The contrast between this article of Prof. Pajot's and the one which he criticizes is striking. One is scientific, the other practical. Dr. Campbell, by experience, arrived at a result that science had failed to reach. The immunity of a woman in labor from the dangers of chloroform Dr. Campbell's experience led him to ascribe to the violent contractions that forced the blood into the brain. This is a conclusion that is not easily attacked.

Prof. Pajot's writing is brilliant, scientific, and is taken as a defence of the position of the French school against the use of anæsthetics in labor. Not a defence, to quote his own words, "by the voice of the most obscure of its professors," but one which will probably have little tendency to interfere with the actual practice of using chloroform in natural labor.

C. C. MATTESON, M.D.

PARIS, April 5th, 1875.

